# Crystalline Lens Staining with Intracameral Phenylephrine During Cataract Surgery

**DOI:** 10.18502/jovr.v16i1.8261

**Published:** 2021-01-20

**Authors:** Michael Tsatsos, Ioannis Athanasiadis, Corrado Gizzi, Balal Shafi, Marilita Moschos, Anant Sharma

**Affiliations:** ^1^Aristotelian University of Thessaloniki, Thessaloniki, Greece; ^2^Moorfields Eye Hospital, London, United Kingdom

##  PRESENTATION

**Figure 1:**
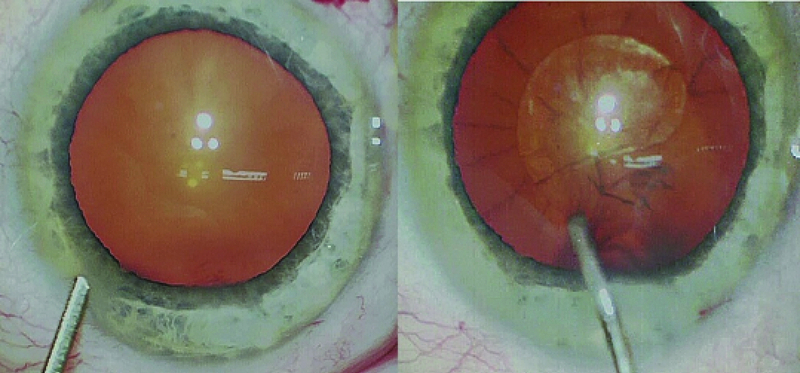
Spike-like staining of the crystalline lens (arrows) following intracameral injection of phenylephrine hydrochloride.

During intracameral use of low concentration phenylephrine in several consecutive routine phacoemulsification cases, we have consistently observed a “spikes” pattern of staining in the crystalline lens structures; however, it was not entirely clear whether this involved just the capsule, the cortex, or both (Figure 1). Although it appears that the staining occurs largely at the level of the anterior capsule, some very faint staining could possibly be seen on the anterior lenticular surface as well. The formulation of intracameral phenylephrine (MinimsⓇ Phenylephrine Hydrochloride, Bausch & Lomb UK Ltd.) that is routinely used in our practice consists of 0.5 ml of 10% phenylephrine preservative free minims mixed with 0.5 ml of 2% lidocaine and 1 ml of Balanced Salt Solution with adrenaline. This mixture (0.2 ml) was injected in the anterior chamber.

**Figure 2:**
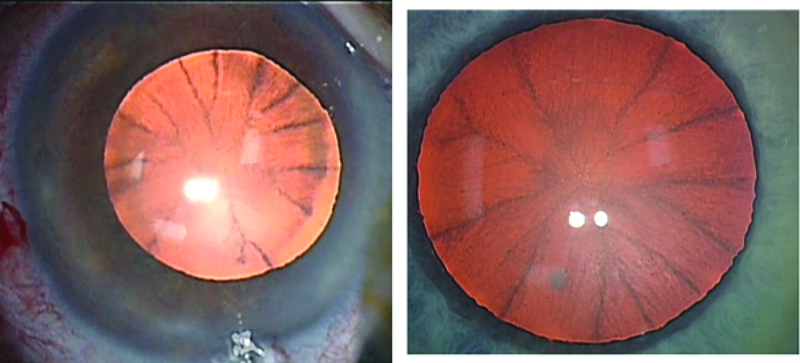
Early (left) and late (right) staining of the crystalline lens following intracameral use of phenylephrine hydrochloride in two different patients.

##  DISCUSSION

Phenylephrine is an α-adrenergic agonist regularly used as a dilating agent in the form of eye drops prior to intraocular surgery such as cataract surgery. It is also frequently used as an intracameral injection in conditions such as floppy iris syndrome to assist with pupillary dilation as well as to increase the iris tone.^[1,2]^

No evidence of capsular staining was observed in our patients at postoperative visits. There were no reported or observed cases of toxic anterior segment syndrome or other systemic or vision-threatening complications intraoperatively or during the postoperative period. Although Lockington *et al* reported the possibility of toxicity associated with the presence of free radicals in intracameral phenylephrine formulations, we report no relevant deviations from routine practice in our patients.^[3]^

Lens staining was consistent in all cases where the intracameral phenylephrine formulation was used. It began to appear in 20 sec, peaking at around 1 min after the intracameral injection (Figure 2). We believe that the resulting appearance of the crystalline lens can facilitate capsulorrhexis in routine as well as in cases of borderline visibility where usually a staining agent such as trypan blue is considered by the surgeon. Thus, no extra provisions need to be made resulting in reduced cost of surgery as well as less logistical burden on the operation theatre.

Furthering our understanding on the cause of crystalline lens staining related to intracameral phenylephrine and its implications will hopefully enable us to use this agent more effectively as a mydriatic and to facilitate capsulorrhexis in routine as well as complicated cases.

##  Financial Support and Sponsorship

Nil.

##  Conflicts of Interest

There are no conflicts of interest.
